# Editorial: Bioinformatics of Genome Regulation and Systems Biology

**DOI:** 10.3389/fgene.2020.00625

**Published:** 2020-07-28

**Authors:** Yuriy L. Orlov, Ancha V. Baranova

**Affiliations:** ^1^Institute of Digital Medicine, I.M.Sechenov First Moscow State Medical University (Sechenov University), Moscow, Russia; ^2^Life Sciences Department, Novosibirsk State University, Novosibirsk, Russia; ^3^Agrobiotechnology Department, Agrarian and Technological Institute, Peoples' Friendship University of Russia, Moscow, Russia; ^4^School of Systems Biology, George Mason University, Fairfax, VA, United States

**Keywords:** genomics, bioinformactics, systems biology, plant science, gene expression, special issue

This Research Topic presents the studies in the field of computational genomics. These papers were discussed at BGRS\SB-2018 (Bioinformatics of Genome Regulation and Structure Systems Biology) multi-conference, along with the hybrid wet-lab/computational genetics studies focused on genome-wide gene expression regulation. The BGRS is the major event in the computational biology field, which has been held in Novosibirsk, Russia biannually since 1998. The main conference is typically followed by a series of special post-conference journal issues covering contemporary computational genetics and genomics applications (Orlov et al., 2016, 2019a; Tatarinova et al., 2019). First Special Issues covering BGRS\SB conference were presented in the *Journal of Bioinformatics and Computational Biology* in 2012 (Kolchanov and Orlov, 2013; Orlov et al., 2015, 2019b) and other platforms (Chen et al., 2017; Baranova et al., 2019; Orlov, 2019; Medical Genetics and Bioinformatics special issue). Starting in 2018, extended discussion of the conference materials in genetics and genomics is being presented in *Frontiers in Genetics*.

In this Research Topic, we arranged the papers by areas of applications—clinical bioinformatics and human genome studies are followed by the plant genetics and then by systems biology applications.

Bah et al. comprehensively reviewed genomics tools and databases allowing us to dissect the pathophysiology of bacterial and parasitic infection, spanning the species from *Mycobacterium tuberculosis* to *Plasmodium falciparum*. These databases provide the data and tools for in-depth investigations of disease outbreaks and pathophysiological mechanisms, genomic variation and co-evolution of hosts and pathogens, diagnostic markers and vaccine targets, with special attention to the contributions of genomics and bioinformatics to the management of both common and neglected tropical diseases, including tuberculosis, dengue fever, malaria, and filariasis.

The TCGA (The Cancer Genome Atlas) database was mined from an entirely new technical viewpoint of developing reference genes with stable mRNA levels for quantitative PCR studies of cancer cells (Krasnov et al.). A scoring system for the assessment of gene expression stability allowed authors to highlight previously untried reference gene candidates, specific to each cancer type, along with several more “universal,” pan-cancer reference gene candidates, namely *SF3A1, CIAO1*, and *SFRS4*. The application on colon adenocarcinoma was presented in Fedorova et al. (2019), another work in the frames of BGRS SB conference series.

The study by Ivanov et al. highlighted methodological problems for an up-and-coming biomarker mining technique, a sequencing of cell-free DNA (cfDNA) in human plasma. As fragmentation patterns of cfDNA are far from being random due to nucleosome patterns reflecting tissue-specific epigenetic signatures, these patterns may be used for guiding the design of amplicon-based NGS panels. Therefore, the sensitivity of mutation detection in liquid biopsy samples may be much improved, allowing for a lessening of the amount of body fluids collected from patients.

Khatun et al. work in the medical bioinformatics field; they have developed a computational tool PreAIP (Predictor of Anti-Inflammatory Peptides), aimed at augmenting the search for novel biologics. Integrative analysis of stomach carcinoma samples by pairing DNA methylation patterns with gene regulatory network topology was presented in Wu et al.. The authors showed conservation of epigenetic patterns across various stages of this important type of human malignancies.

Gene expression regulation at genome level is important in evolution and adaptation studies (Ponomarenko et al., 2017; Igoshin et al.). Igoshin et al. looked into the adaptation of humans to cold climate. They have concentrated on the *TRPM8* gene, which encodes for a cold-sensing ion channel. In a population data set, they found a very promising single nucleotide polymorphism rs7577262 with a signature of selective sweep. Chadaeva et al. employed bioinformatics to discern behavioral pattern in mice and identify variants contributing to the dominance and the subordination traits continuing bioinformatics behavior studies in laboratory animals (Bragin et al., 2017). Using the prediction on-line tool SNP_TATA_Comparator (Ponomarenko et al., 2017) a set of candidate SNP markers contributing to the dominance and the subordination were uncovered. The studies using same SNP analysis tool were continued in Oshchepkov et al. (2019) and Ponomarenko et al. (2020).

Zverkov et al. considered a problem of genome reduction in primitive parasites. Among the two groups of microscopic parasitic invertebrates, the Dicyemida, and Orthonectida, overall morphological organization is much simplified, with tissues and organs almost absent. In these species, homeodomain transcription factors, G-protein-coupled receptors, and many other protein families have undergone a massive reduction. Interestingly, it seems that the dramatic simplification of body plans in dicyemids and orthonectids has evolved independently.

Das et al. discuss the application of ancestry informative markers (AIMs), previously developed for the inference of genomic ancestry in humans (Das and Upadhyai, 2018), for the delineation of gorilla lineages. Three of the four AIMs-determining approaches were successful for gorilla species (Das et al.).

The next group of papers in the Research Topic highlight the findings in genome regulation related to plants genetics. Kovalev et al. developed a computer pipeline and a machine learning classifier of deleterious coding mutations in agricultural plants, with the performance exceeding that of the popular PolyPhen-2 tool. The novel tool will improve the annotation of genes located in QTL and GWAS hit regions. This work was initially discussed at BGRS\SB-2018 plant biology session as well (Orlov et al., 2019c).

Zhang et al. studied abiotic stress in a model of *Populus euphratica* and its sister species *P. pruinosa*, differing by their adaptability to the content of salt in the soil. The authors performed transcriptome analyses of three seed germination phases from both of the species of desert poplar, and presented their findings in a form of a database suitable for use by poplar breeders. Wang et al. also studied *Populus euphratica*, in this case to infer genetics mechanisms of crossover Interference. Four-point linkage analysis allowed them to show the distribution of the crossover interference through the entire genome of this tree, uniquely suited for survival in saline deserts.

The following work by Khassanova et al. continues the line of studies of salinity resistance by exploring expression profiles in the chickpea (*Cicer arietinum* L.). They have tested six accessions of Chickpea ecotypes, all selected from field trials, for tolerance to abiotic stresses, found the involvement of *CaRabC* gene and developed markers for genotyping chickpea germplasm. Gene expression patterns in bread wheat exposed to drought were studied in Zotova et al.. The authors' team had identified general transcription repressor *TaDr1*, a part of *TaDr1, TaDr1A* and *TaDr1B* gene set, with drought-dependent variable expression. It seems that the general transcription repressor *TaDr1* controls expression of *TaVrn1* and *TaFT1* and, consequently, flowering time. These finding have direct implications for plant productivity in the dry environment.

Flowering time in plants is important agricultural feature determined by genetics and environment. Gursky et al. dissected the core genetic regulatory network canalizing the flowering signals to the decision to flower. While discovered and extensively studied in the model plant *Arabidopsis thaliana*, the flowering model may hold in other species (Kozlov et al., 2019). When the authors built a model gene network in chickpea (*Cicer arietinum*), activation from the *FLOWERING LOCUS T* gene or its homologs to the flowering decision led to a high expression of the meristem identity genes, including *AP1*. Different levels of activation from *AP1* may explain the differences observed in the expression of the two homologs of the repressor gene *TFL1* in species compared. Zhao et al. worked on tea plant (*Camellia sinensis*). In this plant, the development of new sprouts directly affects the yield and quality of the tea leaves, by affecting the content of catechins, theanine, and caffeine. Using High-Performance Liquid Chromatography-Mass Spectrometry, authors showed that conserved miRNA are playing a role in primary metabolism of a tea plant during sprouting. Li et al. presented their study of the chloroplast genomes of *Vicia sepium*, an important wild resource plant suitable for cultivation in extreme cold and dry conditions. The authors have compared a new complete chloroplast genome of *V. sepium* with the chloroplast genomes from related genera belonging to tribe Fabeae, then reconstructed the evolutionary history of the chloroplast genomes in these species.

Orlov M. et al. have studied promoters of *Mycoplasma gallisepticum*, an intracellular parasite affecting the respiratory tract of poultry, and found that the vlhA promoters differ by carrying a variable GAA repeats region upstream of transcription start site. These data have implications for the studies of the phase variation in *M. gallisepticum*. The computer technique of such promoter studies were continued in Orlov and Sorokin (2020).

Liu et al. presented their study of gender differences in solitary parasitoid species *Brachymeria lasus*, which has been evaluated as a potential candidate for release to control the gypsy moth, *Lymantria dispar*, a pest of worldwide importance. Work by Qin et al. considers the polyploidy problem in vertebrates. They have analyzed genome organization in the autotetraploid of the red crucian carp (*Carassius auratus* red var.). The loss of chromosomal loci, base variations in non-transcribed spacer, and array recombination of repeat units have been detected.

Overall, we are proud of the Research Topic at Frontiers in Genetics we collated. We hope that you will find this paper collection a stimulating reading, and will consider coming to the next BGRS\SB conferences in Novosibirsk, Russia as well as read next “Bioinformatics of Genome Regulation” Research Topic in *Frontiers* (https://www.frontiersin.org/research-topics/14266/bioinformatics-of-genome-regulation).

## Author Contributions

YO and AB organized the Research Topic as guest editors, supervised the reviewing of the manuscript, and wrote this Editorial paper. All authors contributed to the article and approved the submitted version.

## Conflict of Interest

The authors declare that the research was conducted in the absence of any commercial or financial relationships that could be construed as a potential conflict of interest.
